# ﻿Global population genomics redefines domestication and clinical diversity in the *Aspergillus flavus–oryzae* complex

**DOI:** 10.3897/imafungus.16.172343

**Published:** 2025-12-23

**Authors:** Walter P. Pfliegler, Bálint Németh, Veronika Bodnár, Tünde Pusztahelyi, Ignazio Carbone, István Pócsi

**Affiliations:** 1 Department of Molecular Biotechnology and Microbiology, Institute of Biotechnology, Faculty of Science and Technology, University of Debrecen, H-4032, Egyetem tér 1, Debrecen, Hungary; 2 Doctoral School of Nutrition and Food Sciences, University of Debrecen, H-4032, Egyetem tér 1, Debrecen, Hungary; 3 Central Laboratory of Agricultural and Food Products, Faculty of Agricultural and Food Sciences and Environmental Management, University of Debrecen; H-4032 Böszörményi str. 138. Debrecen, Hungary; 4 Center for Integrated Fungal Research, Department of Entomology and Plant Pathology, North Carolina State University, Raleigh, NC 27695, USA; 5 Food Chain Safety Laboratory Directorate, National Food Chain Safety Office, H-1095 Mester str. 81, Budapest, Hungary

**Keywords:** Aspergillosis, biocontrol, microbe domestication, mycobiome, plant pathogen, traditional fermentation

## Abstract

*Aspergillus flavus* is a globally important human pathogen and agricultural contaminant, while its domesticated relative *A. oryzae* is widely used in food fermentation and biotechnology. Despite their importance, the evolutionary relationship, population structure and domestication history of these fungi remain unresolved. Here, we present the first global population genomic analysis of 639 *A. flavus* and *A. oryzae* isolates from clinical, environmental and food-fermentation sources across multiple continents. Our analyses reveal a complex evolutionary landscape comprising well-separated clades interspersed with highly admixed mosaic groups and potential evidence for multiple independent domestication events giving rise to *A. oryzae*. Clinical *A. flavus* isolates are distributed across several clades and mosaic groups, some overlapping with fermentation strains, highlighting an apparent role of domestication and admixture in shaping pathogen diversity. These results challenge current species boundaries and provide a framework for understanding evolutionary history, taxonomy and pangenomic architecture in these fungi, with broad implications for pathogenicity, food safety, biocontrol and metagenomic surveillance.

## ﻿Introduction

Fungi play diverse, significant and sometimes contrasting roles in agriculture, the food-chain and human health and disease (Hyde et al. 2018; Han et al. 2024; Hill and Round 2024). Despite the immense diversity of the Fungal Kingdom, it is quite rare for a single genus to be both widely used in food production and regarded as a major pathogen of plants and humans. The species-rich *Aspergillus* genus (Houbraken et al. 2020) is probably the most widely known and studied example of such a fungal taxon: its so-called section Flavi (Han et al. 2024) includes, amongst others, two widely known, closely-related and often hard-to-distinguish taxa: *A. flavus*, described by Link in the beginning of the 19^th^ century (Link 1809) and *A. oryzae*, described at the end of the 19^th^ century (Korschelt 1878; Cohn 1884), originally as *Eurotium oryzae*.

The species *A. flavus* is an important crop and post-harvest pathogen with wide-ranging effects on the global food chain, not just due to yield loss, but as the producer of the aflatoxins B_1_, B_2_ and G (AFs), indol-tetramic acid and cyclopiazonic acid (CPA) (Horn 2003). Aflatoxin is significant due to its high toxicity and potent hepatocarcinogenic effects (Peles et al. 2019; Rushing and Selim 2019). *A. flavus* is also the second most important *Aspergillus* species causing human infections, following *A. fumigatus* (Dabas et al. 2022; Kanaujia et al. 2023). However, its non-aflatoxinogenic strains have been found to be effective biocontrol agents against aflatoxin-producing strains (Khan et al. 2021; Molo et al. 2022). The most well-studied such biocontrol agents are Afla-Guard (strain NRRL 21882) and AF36 Prevail (AF36, strain NRRL 18543), used in North America, that competitively interfere with native aflatoxigenic strains (Dorner 2004), while new strains and mixes of strains are also constantly being tested in various geographical settings including Asian (e.g. Rasheed et al. (2024); Xu et al. (2025)), African (e.g. Augusto et al. (2024)) and European countries (Alvarez et al. 2022).

On the other hand, *A. oryzae* is an important microbe in traditional and modern food technology, used in the solid-state fermentation steps of producing soy sauces, fermented soybean pastes, rice wines, baiju and various rice vinegars (Bal et al. 2016; Yun et al. 2020; Chin et al. 2024; Peng et al. 2025), taking advantage of its high hydrolytic enzyme activities. Its most common colloquial name is the koji fungus, originating from the *yellow kōji* of Japanese sake brewing. Its use has recently expanded into biocontrol of both insects and toxigenic fungi (e.g. Alshannaq et al. (2018); You et al. (2024)), postbiotics and animal feed supplements (Seidler et al. 2024) and commercial enzyme production, recently by invoking the toolset of synthetic biology as well (Sun et al. 2024).

These two closely-related species and the problems of their delimitation have generated interest since the advent of molecular genetic techniques. Based on early molecular genetic findings, the koji fungus was reclassified as a variety of *A. flavus* (Kurtzman et al. 1986). The single-species concept for the two fungi was not accepted in a recent large-scale revision of section Flavi by Frisvad et al. (2019). However, the authors note that *A. oryzae* and *A. sojae* appear to be domesticated forms of the aflatoxigenic species *A. flavus* and *A. parasiticus*, respectively. In line with this, the origin of *A. oryzae* from within the species *A. flavus* through domestication has generally been supported by the literature of the past decades, yet the species status of both is upheld (Chang et al. 2006; Gibbons et al. 2012; Watarai et al. 2019; Han et al. 2024; Jeong and Seo 2025). The regulatory confusion that conspecificity would generate is often cited as a pragmatic reason to maintain their separate species status (Geiser et al. 1998; Houbraken et al. 2014). A few analyses have challenged the view of an extremely close relationship between the two “species”. For example, Kjærbølling et al. identified *A. minisclerotigenes* and *A. aflatoxiformans* as the closest relatives of the koji fungus (Kjærbølling et al. 2020).

The genome of *A. oryzae* was one of the first in the genus to have a full assembly (Machida et al. 2005), while a chromosome-level assembly of *A. flavus* strain NRRL 3357 has recently been published (Skerker et al. 2021) as well. Recent advances in genome sequencing technology have enabled the sequencing and analysis of hundreds of strains and isolates, resulting in significant insights into the population structure and genome evolution of *A. flavus* and *A. oryzae* (Moore et al. 2017; Gell et al. 2020).

In the case of *A. flavus*, isolates were first distinguished by sclerotial morphotypes into the S (small sclerotia; should not be confused with another species characterised by small sclerotia, namely *A. minisclerotigenes*) and L (large sclerotia, with high variation on toxigenicity) groups and some small sclerotial isolates from Thailand were described as separate variant of the species, as A. flavus var. parvisclerotigenus (Saito and Tsuruta 1993). Later, based on phylogenetics of a limited number of sequenced genes, groups I (S- and L-strains that produce AFB1) and II (S-strains that produce AFB1 and/or G-aflatoxins) were distinguished (Geiser et al. 1998). Importantly, *A. oryzae* strains fell into group I. Group I was subsequently divided into three lineages: IA, IB and IC (Geiser et al. 2000). Group IB was associated with *A. oryzae* strains, while the sclerotial morphotypes did not cluster with the newly-established genetic groups, as both group IA and group II included strains with the S morphotype. Later works using phylogenetic methods revealed the importance of recombination events amongst the lineages and chemotypes (aflatoxigenic and non-aflatoxigenic types) of group I (Moore et al. 2009, 2013). Moore et al. (2017) examined a worldwide collection of 1,304 *A. flavus*, *A. oryzae*, *A. nomius*, *A. parasiticus* and *A. sojae* isolates with multi-locus sequence typing and identified seven distinct genetic clusters. *A. flavus* was grouped into three clusters: L-type (group I), S_B_ and S_BG_ (group II). The latter two represent distinct chemotypes of S-type isolates, producing only aflatoxin B (S_B_) or both aflatoxins B and G (S_BG_). The S_BG_ cluster was proposed to be of hybrid origin, containing haplotypes from both *A. flavus* and *A. parasiticus*. The first study to compare three S-type and three L-type genomes of the species also found no clear phylogenetic separation between the two morphotypes (Ohkura et al. 2018).

In a study of 94 *A. flavus* whole-genome sequences, primarily from the continental United States, Drott et al. (2020) identified three genetically distinct populations of L-type isolates (A, B and C), based on phylogenomic networks, along with a fourth, then-unnamed group comprising two S-type isolates. Population A was characterised by relatively frequent sexual processes, based on linkage disequilibrium decay analysis and higher aflatoxin production. Population B had variable aflatoxigenicity and included non-aflatoxigenic isolates and was determined to be sympatric to population A. Population C was mainly non-aflatoxigenic (Drott et al. 2020). The genomes were later used in a study describing the secondary metabolic gene clusters in the pangenome of the species (Drott et al. 2021) and in a phylogeny for twelve newly-sequenced isolates from Pakistan (Ajmal et al. 2022). The latter work recovered the same three populations. Gangurde et al. (2024) inferred a phylogeny of 346 *A. flavus* isolates, including 225 newly-sequenced ones from the US and recovered the same populations, with a focus on the pangenome of the species. Most recently, 70 predominantly European clinical isolates were sequenced and phylogenomic analysis revealed a fourth population greatly enriched in clinical isolates, named Population D (Hatmaker et al. 2025). The group of small sclerotial isolates was also recovered and designated as a population of its own, named S-type. A recent study by Molo et al. (2022) utilised a ddRADseq-based dataset of 815 samples, which were subjected to phylogenetic, admixture and cluster analyses. The authors reported that population A from Drott et al. (2020) largely corresponds to lineage IC, population B to lineage IB, while population C showed affinities to IC. Based on these results, two distinct evolutionary lineages, IB and IC, were delineated (lineages IA and II strains were not included in this analysis). Lineage IB was genetically more homogeneous, whereas lineage IC exhibited a highly reticulate pattern in the neighbour-net analysis and greater variation in the SNP-based principal component analysis. Notably, using 353 unique genome-wide haplotypes, Molo et al. (2022) also demonstrated that SNP-based analyses were largely unaffected by changing the reference genome from *A. flavus* NRRL3357 to the *A. oryzae* RIB40 assembly, providing further evidence of the extremely close relatedness between the two taxa.

In the case of *A. oryzae*, eight main clades have been described and designated by letters A – H based on a large-scale analysis of 82 genomes (Watarai et al. 2019) with evidence of past recombination events amongst clades. The clades were also recovered in a subsequent analysis of 91 genomes (Chacón-Vargas et al. 2021). It is notable that the *A. oryzae* literature typically refers to “clades”, whereas *A. flavus* is more often described in terms of lineages or groups (IA, IB, IC and II) or populations (A, B, C, D and S-type) in literature, as outlined above. Most other members of section Flavi still lack comprehensive population-level genome sequencing studies (Kjærbølling et al. 2020).

Despite the steadily increasing number of available assemblies and whole genome sequencing projects, only recently has a phylogenomic analysis been published that included a considerable number of genomes from both species. Han et al. (2024) highlighted the genomic similarities between the two *Aspergillus* species and recovered *A. oryzae* as a clade nested within the genetic diversity of *A. flavus*. However, the phylogeny, based on amino acid sequences of all single-copy orthologous genes in 93 assemblies, did not include the *A. flavus* populations of Drott et al. (2020) or the previously described lineages I and II.

Despite the recent advancements in the phylogenomics of these *Aspergillus* species, clades of *A. oryzae* and populations and lineages of *A. flavus* have not been directly compared. Additionally, the use of the *A. flavus* and/or *A. oryzae* reference genomes is not consistent in the literature. There is, thus, a need for more robust taxonomy of these important fungi as proposed earlier (Arias et al. 2021). Our aim in this study was to collect available short-read sequencing data on these two species, summarise information on the strains and isolates they represent and subject them to phylogenomic and population genomic analysis. Our focus in this is on the phylogenetic relationships of clades and potential mosaic groups, and their geographic distribution.

## ﻿Materials and methods

### ﻿Overview of taxonomy, literature and databases

To place phylogenomic and population genomic findings into both a taxonomic and applied context, we reviewed: (1) literature on the taxonomic descriptions and type strains along with described infraspecific taxa of the two species; (2) their population structures, clades, lineages and assemblies; and (3) as well as published BioProjects with sequences. We also queried major fungal taxonomic databases (Index and Species Fungorum, Mycobank) and databases frequently used in molecular genetic species identification (NCBI Taxonomy Database, FungiDB, SILVA or UNITE) to check the status of *A. flavus* and *A. oryzae* and infraspecific taxa associated with them.

### ﻿Novel isolates and isolation of genomic DNA

Genomic DNA was isolated from three Hungarian isolates from the collection of the Central Laboratory of Agricultural and Food Products (Faculty of Agricultural and Food Sciences and Environmental Management, University of Debrecen) and our laboratory’s stock of the reference strain NRRL 3357 as a control strain for genomic analyses. Library preparation was performed using tagmentation with the Nextera DNA Flex Library Prep kit (Illumina, San Diego, CA, USA) according to the manufacturer’s protocol. Sequencing was performed using 150 bp paired-end reads on an Illumina NextSeq 500 system, with approximately 140× coverage of the nuclear genome. Raw reads were deposited in NCBI SRA under BioProject PRJNA1307175. Accession numbers are listed in Suppl. material 2.

### ﻿Published genomes

A comprehensive list of published genomes sequenced using short-read technology was compiled for both species and additional genomes were identified by searching NCBI BioProjects with the species’ names (accessed 16 January 2025). Metadata associated with the sequencing files were collected from ENA and SRA and information on the isolates and strains was compiled from publications, including an ontology of habitats. Habitat categories included Agriculture (with subcategories of plant types or field soil), Environmental (e.g. soil samples where agricultural origin was not specifically stated), Human (clinical isolates) and Food industry (e.g. koji, meju, nuruk or qu). Illumina sequencing runs were downloaded from SRA. The Illumina FASTQ sequencing files were trimmed and filtered using fastp for further analysis (Chen et al. 2018). In the list of genomes, geographic areas were grouped into larger categories following the United Nations geoscheme (accessed 01 June 2025), while isolation sources were grouped into the following major categories: Environmental, Agriculture, Human, Food fermentation and Artificial Environment.

### ﻿Phylogenomic analysis

Illumina reads were mapped to the ASM901741v1 (GenBank accession: GCA_009017415.1) *A. flavus* strain NRRL 3357 (Skerker et al. 2021) reference genome using BWA 0.7.17. (Li and Durbin 2010). As the reference contained no mitochondrial assembly, the complete mitochondrial sequence of the isolate CA14 (GenBank accession: CP061812.1) was appended to the file. It has been demonstrated that the two commonly used assemblies in section Flavi are interchangeable (Molo et al. 2022), thus, *A. oryzae* genomes were also mapped to this former *A. flavus* reference. Sorted BAM files from reference-based alignments were obtained using Samtools 1.7 (Li and Durbin 2010) and Picard-tools 2.23.8 was used to mark duplicated reads (Van der Auwera et al. 2013). BAM files were subjected to local re-alignment around indels and joint variant calling using GATK 4.1.9.0. (Van der Auwera et al. 2013; Poplin et al. 2018) with ploidy set to 1. In the first step, genomic VCF files were obtained with GATK Haplotype Caller and joint genotyping of the gVCF files was applied. Subsequent filtering of resulting VCF files was based on either SNPs or INDELS. SNPs were filtered according to the following parameters: QD < 5.0; QUAL < 30.0; SOR > 3.0; FS > 60.0; MQ < 40.0; MQRankSum < -12.5; ReadPosRankSum < -8.0. INDELS were filtered according to the parameters QD < 5.0; QUAL < 30.0; FS > 60.0; ReadPosRankSum < -20.0. INDELS were then left-aligned. For the final VCF files, INDELS and SNPs were merged, filtered and non-variant sites were removed. Calling was performed for every chromosome and the mitochondrial genome separately. Two outgroup genomes were also included in the cohort variant calling workflow: *A. minisclerotigenes* E1406 and *A. parasiticus* AA-M17-157. Initial tests on the dataset (SNP calling as described here and neighbour-net graphs as described below) showed that a few *A. flavus* genomes from literature (Arias et al. 2020) clustered with *A. minisclerotigenes* and these were excluded from the final dataset (samples E1288, E1293, E1316, E1376). The initial analyses also showed that mapping the *A. minisclerotigenes* and *A. parasiticus* reads to the NRRL 3357 reference results in a high proportion of callable sites despite their divergence (gap and ambiguity: 9.36% and 14.22% of total 3,469,698 called variant sites, respectively). Thus, they are appropriate as outgroups in the SNP matrix-based analyses.

The SNP VCF files from cohort calling were used to produce genotype matrices using vcf2phylip (Ortiz 2019). Matrices were used as input in the software SplitsTree 4.15.1. (Huson and Bryant 2006) to create a phylogenomic neighbour-net graph using uncorrected P-distance. This was also converted to a neighbour-joining tree and visualised in iTOL, along with additional data (e.g. population genomics, see below) (Letunic and Bork 2019). Linkage disequilibrium pruning was not applied and, therefore, all SNPs were considered for building the network. The neighbour-net graph was generated both with and without the outgroup species to improve the visibility of the resulting figures.

### ﻿Identity-by-state (IBS) analysis

IBS analysis was carried out using SNP data with the R package “SNPRelate” 1.24.0 (Zheng et al. 2012) using the function “snpgdsIBSNum” with default settings, calculating the minor allele frequency and missing rate for each SNP over all the samples. The analysis was based on converting the input VCF file with the snpgdsVCF2GDS function and creating n-by-n matrices. Due to the method used in the package (.gds files are converted from .vcf files), only biallelic SNPs were kept. Since linkage disequilibrium pruning was not applied for this analysis, all 1,764,492 biallelic SNPs were considered. SNPRelate expects diploid calls, thus the genotypes in the vcf file were corrected as if they were diploid homozygous calls using awk. This way, two informative datasets were obtained: the number of identical genotypes between each pair of genomes (analogous to IBS2 of diploids) and the number of non-identical genotypes (analogous to IBS0 of diploids). Instead of applying clustering, the order of genomes was changed to fit that of the neighbour-joining tree. Results were visualised with the R package “heatmaply” (Galili et al. 2018) with default settings. Zoomable .html files for heatmaps are uploaded to FigShare (doi: 10.6084/m9.figshare.29896676). After clades and the genomes belonging to them had been established, the table-format data generated by the IBS analysis was used again, to calculate SNP identity amongst all possible pairs of genomes in each pair of groups. These were visualized as violin plots for all possible group comparisons (StatisticsKingdom 2017). Outgroup species were excluded from this analysis.

Population genomic analysis was performed using fastStructure 1.0 (Raj et al. 2014) run on SNPs filtered for biallelic variants with minor allele frequency greater than 5%. Linkage disequilibrium pruning was conducted with plink 1.9 (Chang et al. 2015) (plink -indep-pairwise 50 1 0.5). Populations were specified from 2 to 10. The chooseK command was used to identify the best number of populations. The population genomic analysis after pruning relied on 850,234 variants (i.e. ~ 48% of all biallelic SNPs). Population genomic data was visualied in iTOL as a dataset added to a dendrogram. Outgroup species were excluded from this analysis.

A subset of the genomes was chosen for Maximum Likelihood analysis using IQTree 2.3.6. (Minh et al. 2020) and ModelFinder (Kalyaanamoorthy et al. 2017). From each clade and mosaic group, five representative genomes were chosen randomly and only these were kept in the genotype matrix. The matrix was used as an input for finding the best model and to obtain a dendrogram with 1000 ultrafast bootstrap approximations, which was visualised in iTOL. A single *A. minisclerotigenes* outgroup (E1406) was used to root the dendrogram.

The above mentioned phylogenomic and population genomic analyses were run on the individual chromosomes and on the mitochondrial genome, as well as on a combined dataset as well, representing the whole genome. However, the *A. oryzae* and *A. flavus* reference genomes are not completely syntenic, therefore, relying on the chromosomal boundaries of the NRRL 3357 genome could introduce uncertainties. Thus, we limited detailed analyses to the combined dataset.

## ﻿Results

### ﻿Neotypes, reference genomes and the status of *A. flavus* and *A. oryzae*

In this work, we first reviewed the original descriptions of *A. flavus* and *A. oryzae*. We also tracked how their taxonomic status changed over time, which neotype strains were assigned to them and which of these has an available genome sequence. *A. flavus* Link was described in the beginning of the 19^th^ century (Link 1809). The first mention of *A. oryzae* is as *Eurotium Oryzae* Ahlburg [sic] in a publication on Japanese sake-brewing dating back to 1878 (Korschelt 1878). In this publication, Korschelt scientifically described the koji fungus in the name and in honour of Ahlburg (Korschelt 1878). Years later, it was later reclassified as *A. Oryzae* [sic] by Cohn (1884), its full botanical name becoming *A. oryzae* (Ahlb.) Cohn. Several variations and forms of *A. oryzae* have been described in the 20^th^ century and many of them have been subsequently synonymised (Raper and Fennell 1973). Mycobank lists 18 such names (Mycobank, accessed 16.01.2025). It is worth noting that the publication of A. oryzae var. basidiferus Costantin & Lucet in the early 20^th^ century (Costantin and Lucet 1905) resulted in the previously described original koji fungus being recognised as A. oryzae var. oryzae (Ahlb.) Cohn, in accordance with the rules of botanical nomenclature.

Fungal nomenclature relies on type strains or herbarium type material deposited in collections and these types must be taken into account when discussing the taxonomic status of the two fungi. Due to *A. flavus* and *A. oryzae* being first described in the 19^th^ century, original type strains for the species *A. flavus* and *A. oryzae* have not been designated and deposited, further complicating their taxonomic status as only neotypes are available. The neotype of *A. flavus* is available in multiple collections (NRRL 1957, ATCC 16883, CBS 569.65, CBS 100927, IMI 124930). Interestingly, this neotype is far from a typical representative of the species: it was collected from the cellophane diaphragm at an unknown location in the South Pacific (ARS Culture Collection, accessed 16.01.2025). Its genome was recently sequenced, but it is not considered the commonly used reference. The widely adopted reference genome is NRRL 3357 (Skerker et al. 2021), which includes complete chromosomes, but lacks an assembled mitochondrial genome.

A neotype for the taxon *E. oryzae* Ahlb., collected in Japan, was designated as IMI 16266 (also deposited as CBS 102.07 and CBS 110.47). Furthermore, another type strain was designated for A. oryzae var. oryzae (Ahlb.) Cohn, which was collected in 1969 in Japan and currently deposited in multiple collections (ATCC 42149, CBS 466.91, IFO 6215, RIB40, WB 5032). The assembled genome of this strain is widely used as the reference genome for *A. oryzae* (Machida et al. 2005).

Major taxonomic databases now regard *E. oryzae* Ahlb. and *A. oryzae* Cohn as a variety of *A. flavus* Link. Its full botanical name is thus listed as A. flavus var. oryzae (Ahlb.) Kurtzman, M.J. Smiley, Robnett & Wicklow. Whenever this infra-specific status is accepted, the non-*oryzaeA. flavus* taxon has to be specified as A. flavus var. flavus. In contrast, the taxon A. oryzae var. oryzae (Ahlb.) Cohn 1884 is accepted as a legitimate and the current name for the koji fungus as it has never been synonymised in literature (Mycobank and Index Fungorum, both accessed 16.01.2025). The NCBI Taxonomic Database and, hence, the NCBI RefSeq database often used for mapping-based metagenomic analysis (e.g. Yan et al. (2024)) (accessed 15.03.2025) accepts the two species as separate, while UNITE (accessed 15.03.2025) lists only *A. flavus* barcode sequences. The FungiDB database (Basenko et al. 2024) and the SILVA ribosomal RNA reference database (Glöckner et al. 2017) (both accessed 15.03.2025) lists both species separately.

### ﻿Combined phylogenetic and population genomics of 639 *A. oryzae* and *A. flavus* genomes re-defines clades, mosaic groups and the origin of the koji fungus

We identified, tabulated and downloaded all available *A. oryzae* and *A. flavus* short-read genome sequences from previously published papers and additional BioProjects containing publicly accessible sequencing files (NCBI SRA, accessed 16 January 2025). Three additional isolates from Hungary were also sequenced in this study and added to the list, which contains 639 genomes from 35 studies and Bioprojects. A small number of genomes above this number uploaded into SRA as S-type *A. flavus* clustered with *A. minisclerotigenes* (E1288, E1316, E1293, E1376, E1406) (Arias et al. 2020) in a preliminary phylogeny. These were discarded from further analyses, except for E1406 which was used as an outgroup, along with the *A. parasiticus* strain AA-M17-157 (Hatmaker et al. 2024, 2025). A strain in SRA described as *A. parasiticus* NRRL 2999 proved to be identical to *A. flavus* NRRL 33757, as shown previously (Fountain et al. 2020; Houbraken et al. 2021) and was included with an identifier ‘NRR33572999’.

A limited number of strains were sequenced by more than one study due to their importance in biocontrol or as a reference genome (Suppl. material 2). Thus, the total amount of unique strains and isolates (excluding outgroups) in the collection presented in this study from the past 20 years amounts to 632, collected from 17 countries and regions.

**Figure 1.**
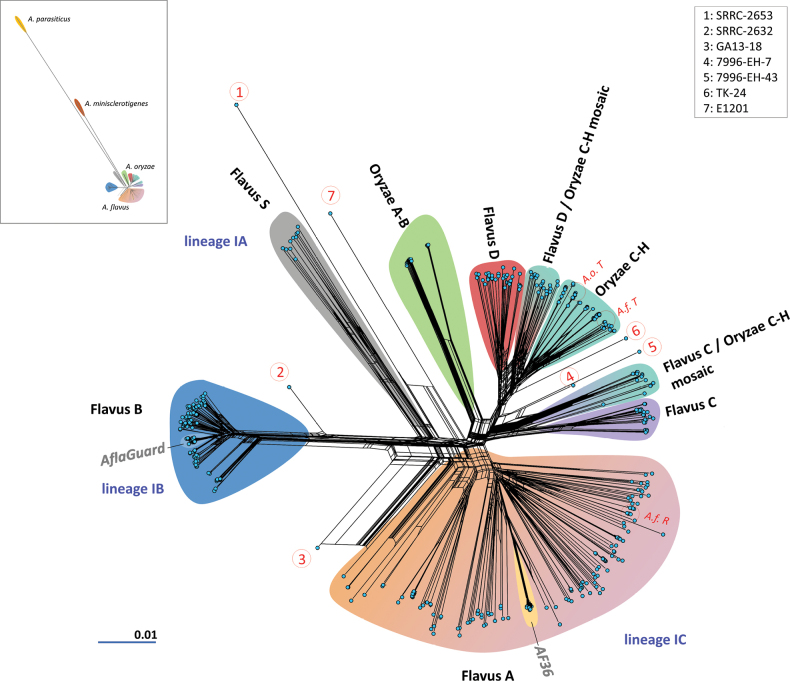
Combined neighbour-net analysis of 639 *A. flavus* and *A. oryzae* genomes, with clades and mosaic groups highlighted with background colours. The network is based on distance data of 1,667,702 parsimony-informative and 1,801,996 singleton sites. Note that, although parallel edges leading to individual samples are also highlighted with background colours, their bases should not be interpreted as the proposed common ancestor of a given clade, the edges merely representing possible alternative evolutionary routes between major groups of genomes. The inset in the top left corner shows the network structure where the two outgroup species are also included. Red numbers in circles represent genomes in singleton positions as explained in the top right corner. Each blue circle is a sample. Important genomes are marked: the neotype of *A. flavus* (*A.f. T*), the neotype and reference genome of *A. oryzae* (*A.o. T*) and the reference genome of *A. flavus* (*A.f. R*). The subclades of the biocontrol Afla-Guard and AF36 strains are highlighted and marked. Clade and mosaic groups are given in black-coloured text, lineages described for *A. flavus* are written in blue text.

The 639 genomes, including the replicates, were included in the variant cohort calling pipeline and in subsequent phylogenomic and population genomic analyses. Based on an SNP-matrix containing 1,667,702 parsimony-informative and 1,801,996 singleton sites (as determined by running IQTree on the whole genotype matrix) from all called variant sites of the genome, we first generated a distance matrix of the genomes and, subsequently, a neighbour-net graph based on this, following earlier studies (Drott et al. 2020; Molo et al. 2022). Using the NRRL 3357 reference genome for all genomes and the approach of reference-based variant calling resulted in a very high proportion of comparable variants. Only four samples had more than 10% gaps and unknown variants across the dataset and the mean percentage of such undetermined sites was 5.45%. Our dataset recovered groups within both *A. flavus* and *A. oryzae*: the “populations” described for *A. flavus* and the clades described for *A. oryzae*. Inclusion of both fungi in a single phylogenomic network enabled us to compare the diversity within groups of the two species. The number of described clades for the koji fungus proved to be too high to enable meaningful comparisons with the more diverse “populations” of *A. flavus*. The described *A. oryzae* clades (Watarai et al. 2019; Chacón-Vargas et al. 2021) were thus grouped into two superclades, from here on referred to as clade Oryzae A-B (former clades A and B) and clade Oryzae C-H (former clades C to H) (Watarai et al. 2019; Chacón-Vargas et al. 2021) (Fig. 1). For the sake of consistency, the “populations” of *A. flavus* are also referred to as clades in our analysis in cases when population genomic analysis does not point to mosaic genomic origin.

The S-type “population” of *A. flavus*, from here on referred to Flavus S clade, had the fewest genomes and formed a distinct group connected by long parallel edges to the rest of the network. This included the genome AF70 and, thus, corresponds to the lineage IA of *A. flavus*. The “population B”, from here on referred to as the Flavus B clade, was also connected by long parallel edges to the rest of the groups and was characteristically compact, suggesting lower diversity. The Flavus B encompassed a subgroup which consisted of the Afla-Guard biocontrol strain and many highly similar, almost indistinguishable field isolates (Fig. 1). This group thus corresponds to lineage IB, based on the genomes clustering into it that are classified into lineage IB in literature. The “population A”, named here Flavus A clade, was characterised by higher diversity, unclear within-clade connections and formed a broad sub-network. This included the AF36 biocontrol strain and several extremely similar field isolates connected to the Flavus A subnetwork by long parallel edges (thus, clear separation and unclear within-group relations due to very high similarity). Based on the list of genomes clustering to this Flavus A clade, it largely corresponds to lineage IC in literature.

**Figure 2.**
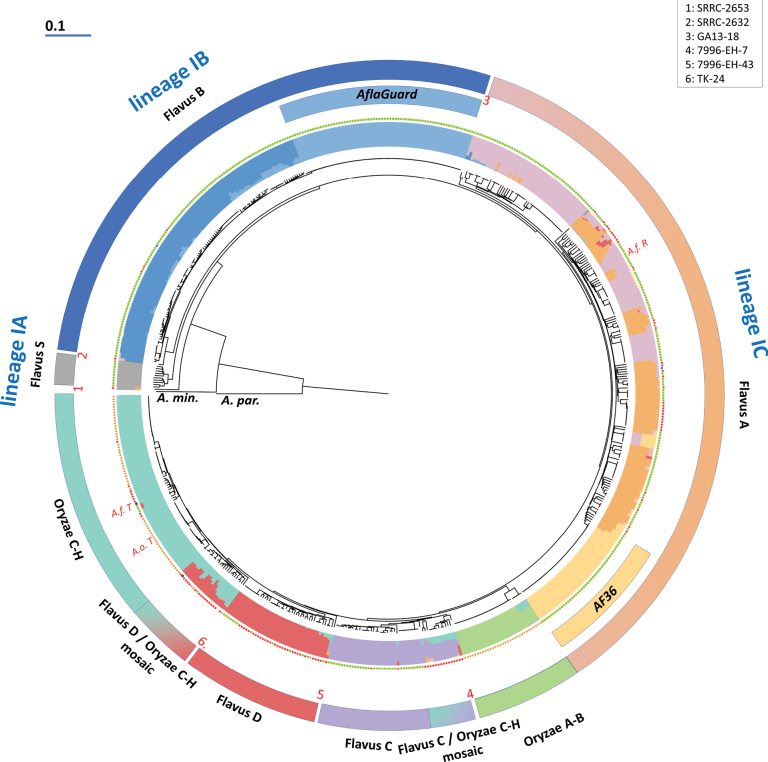
Neighbour-Joining dendrogram of the 639 genomes along with two members of the outgroup species. The dendrogram is based on the same data as the network in Fig. 1 and shows the population genomic data for k = 10 for each sample. On the outside, clade and mosaic group assignment of the given genomes are indicated with colours and clade and mosaic group names (black font) and lineage names (blue font) are shown. Important genomes are marked: the neotype of *A. flavus* (*A.f. T*), the neotype and reference genome of *A. oryzae* (*A.o. T*) and the reference genome of *A. flavus* (*A.f. R*). Red numbers in circles represent genomes in singleton positions as explained in the top right corner. The origin of each sample is coded with pictograms outside the population genomic dataset representation: green dots represent Agriculture origin, orange triangles origin from Food fermentation, red stars originate from Humans, blue circles originate from Nature and black squares originate from Artificial environments.

On the other hand, several described *A. flavus* “populations” could not be clearly assigned to any *A. flavus* lineages described in earlier literature and the *A. oryzae* genomes were also distant from the *A. flavus* lineages. The “populations C and D”, here named Flavus C and Flavus D clades, respectively, both showed close relationship to the Oryzae C-H clade. Furthermore, two more distant groups of isolates, one between the branching of (Flavus D and Oryzae C-H) and one between the branching of [Flavus C and (Flavus D + Oryzae C-H)] was identified (Figs 1, 2), containing a smaller number of isolates that, in most cases, have not been assigned to clades or populations before. Their placement suggested putative mosaic or recombinant genomes, which was subsequently evaluated further, as described below. Finally, six or seven isolates (depending on whether the network included the outgroups or not) were singletons without placement in any subgroup of the network. It must be noted that none of the genomes sequenced by any study and analysed here corresponds to the *A. flavus* lineage II; thus, this entire lineage is absent from these phylogenies and population genomic analyses.

**Figure 3.**
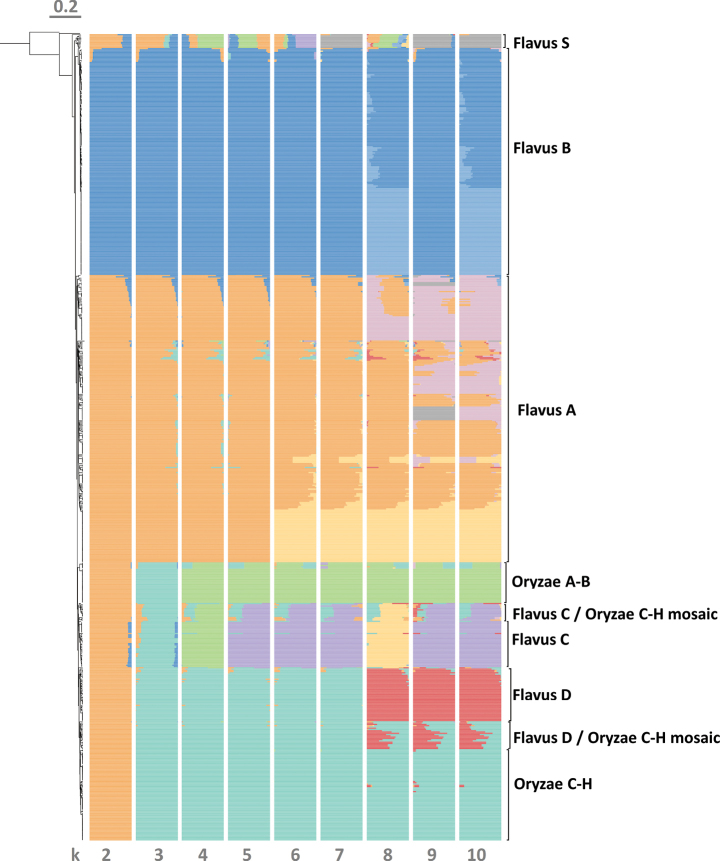
Population genomics with different models ranging from k = 2 to k = 10, based on a linkage disequilibrium pruned dataset. The order of the samples corresponds to the dendrogram in Fig. 2. Population genomic results as stacked bar charts are added to the dendrogram (shown on the left edge). Colour codes in the bar chart reflect to the clades’ colour codes in Figs 1–2. for k = 10. For other k values, clades are not necessarily recovered in proposed populations; thus, colours were chosen to represent the clade that forms the majority of the delineated population at a given k value. Outgroup species were not included in the population genomic analysis.

Based on observations from the phylogenomic network, we integrated fastStructure-based population genomic analyses and a neighbour-joining dendrogram phylogeny to determine the origin of the two newly-observed subgroups and to potentially subdivide the Flavus A clade into well-defined subclades. Based on the network, various numbers of possible populations (in the sense of population genomic analysis) were tested after linkage disequilibrium pruning with the highest k = 10. While the best model pointed to 10 optimal genetic clusters, the populations determined using fastStructure did not fully correspond to the groups of genomes observed on the phylogenomic network, due to two distinct phenomena that can be inferred from a comparison of the neighbour-joining dendrogram (Fig. 2) and the various population genomic models, described below. To facilitate this, data with k = 10 were added to the dendrogram, while data with all possible k values are also shown next to a simplified dendrogram in Fig. 3.

Population genomic analysis recovered the Flavus S clade (lineage IA) as a well-defined population at k = 7, 9 and 10, but as a group of mosaics comprising heterogeneous genetic backgrounds with other k values (Fig. 3), suggesting that the clade is at least partially derived from admixture and subsequent diversification, as the within-clade diversity was substantial (Figs 1, 2). This diversity is also evident when the number of shared variants is compared across all genomes of the clade in a pairwise manner (Suppl. material 1). The number of such shared variants is notably more variable in the Flavus S clade than in other clades.

The Flavus B clade was divided into two populations, of which one corresponded to the Afla-Guard genome and other extremely closely-related isolates from the USA. The other population contained the remaining Flavus B genomes. Thus, the high number of Afla-Guard-like samples resulted in splitting this clade into two distinguishable populations including several samples with evidence of genetic admixture between the two.

The Flavus A clade was split into three populations comprising a high number of admixed samples as well. Two populations showed variable admixture and variable positions on the dendrogram (Fig. 2) and the network (Fig. 1), while the third population corresponded to the AF36 biocontrol strain and its subclade of very closely-related field isolates, similar to what was observed in the Afla-Guard subclade. The differentiation of three populations was consistent with k values from 8 to 10; however, with k = 6 and k = 7 models, two populations were delineated: one corresponding to the AF36 subclade and the other comprising the remaining genomes (Fig. 3). Based on the integration of insights from the network, the dendrogram-based phylogenies and the population genomic data, we determined that the Flavus A clade cannot be meaningfully subdivided into distinct subclades, apart from the AF36 subclade. Further signs of relatively small levels admixture from Flavus D and Flavus B furthermore complicates any subdivision of Flavus A into monophyletic subgroups (Fig. 2). Shared SNPs across pairwise comparisons with members of other clades and groups were also characteristically variable in this clade. Almost all other clades had a high proportion of very similar genomes to some members of the Flavus A (Suppl. material 1).

The Flavus C, Flavus D, Oryzae C-H and Oryzae A-B clades were all recovered as individual populations with most models (including k = 10). In the latter, a small group of isolates showed introgression from Flavus B (including the AflaGuard subclade) (Figs 2, 3). This also resulted in a characteristically bimodal distribution of shared SNPs in pairwise comparisons (Suppl. material 1). The ten populations determined by FastStructure-based population genomic modelling on a linkage disequilibrium pruned dataset did not identify further populations. The two additional observed groups on the phylogeny, namely the group of genomes between the branching of (Flavus D and Oryzae C-H) was found to be a mosaic group, hereafter referred to as Flavus D/Oryzae C-H mosaic and the group between the branching of [Flavus C and (Flavus D + Oryzae C-H)] was shown to contain mosaic genomes predominantly with Flavus C origin, along with variable levels on introgression from Oryzae C-H and/or Flavus A. This mosaic group is named here Flavus C/Oryzae C-H mosaic. The names of these newly delineated mosaic groups were added to the phylogenies (Figs 1, 2) and to the list of genomes in Suppl. material 2. It should be noted that none of the population models recovered *A. flavus* and *A. oryzae* as distinct, monophyletic clades.

**Figure 4.**
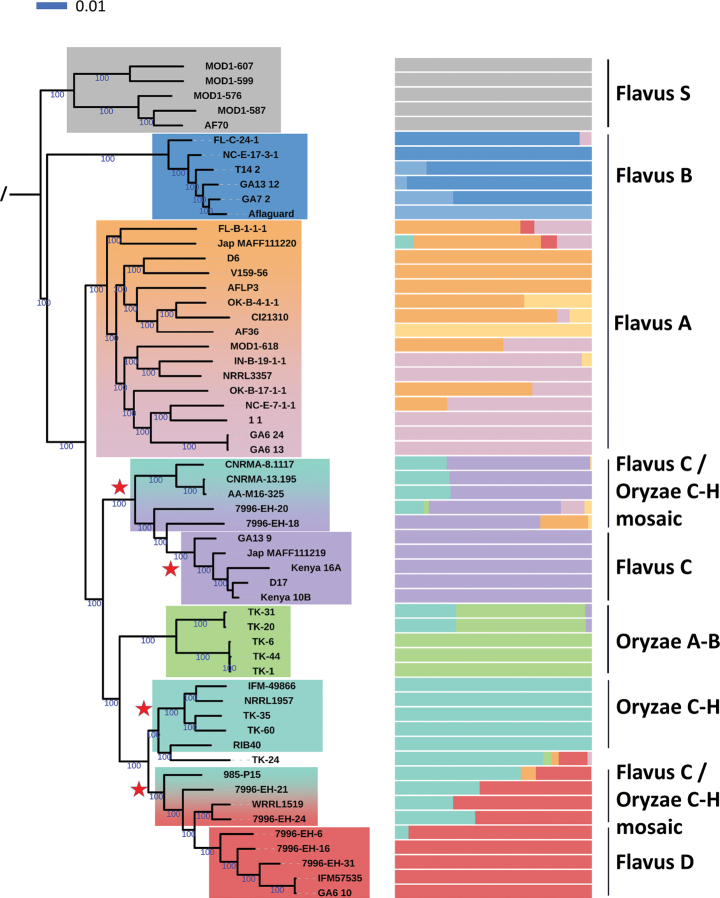
Maximum Likelihood phylogeny for a subset of genomes. Clades and mosaic groups as determined above are colour coded, next to sample names, population genomic data for k = 10 are shown, along with clade and group names. Ultrafast bootstrap support values are added to the branches when significant (> 95%). Red stars mark apparent monophyletic groups that contradict both the NJ network and the population genomic model. The outgroup *Aspergillus minisclerotigenes* isolate has been removed from the phylogeny for visualisation.

We also conducted a Maximum-Likelihood (ML) phylogenomic analysis on a smaller dataset, including at least five genomes representing each clade, subclade, population and mosaic group (the diverse Flavus A was represented by 16 genomes), using 1,000 ultrafast bootstrap replicates (Fig. 4). This phylogeny was based exclusively on the 1,667,702 parsimony-informative variants; however, it exhibited the same general evolutionary patterns as the neighbour-joining network and dendrogram. The Flavus S, B, A, C, D and the Oryzae A-B clades were recovered as monophyletic, with very high (> 95%) bootstrap support. The phylogeny showed the Oryzae C-H clade to include the genome of TK-24, which was found to be in a singleton position in other analyses. The Oryzae A-B clade was also subdivided into an admixed and a non-admixed subclade, as in the neighbour-joining analyses. As described above, the Flavus A clade could not be resolved into monophyletic subclades that would correspond well with population genomic data; various branches with very high bootstrap support in this clade contained genomes with variable levels of admixture amongst the AF36 population and the two other populations in Flavus A.

As expected, the ML phylogeny placed the inter-clade mosaic genomes at various positions. The Flavus C/Oryzae C-H mosaic group was paraphyletic, forming a monophyletic clade only when the Flavus C lineage was included. Similarly, the Flavus D/Oryzae C-H mosaic group was paraphyletic. Thus, ML analysis simplified the positions of the mosaic groups and grouped each with one clade with high bootstrap support, despite the clearly heterogeneous genomic background of some samples, as determined by the FastStructure analysis (Fig. 4). The ML phylogeny resolved four superclades with maximum ultrafast bootstrap support as follows: (Flavus S; Flavus B; Flavus A; (Flavus C, Oryzae A-B, Oryzae C-H, Flavus D)). Thus, the ML analysis, as the NJ algorithm described above, did not clearly separate *A. flavus* and *A. oryzae*.

**Figure 5.**
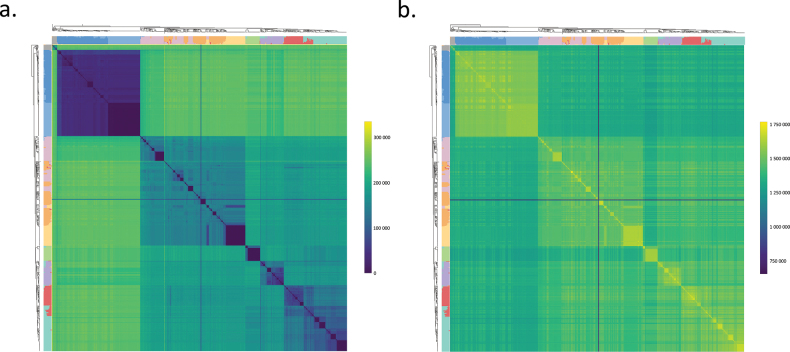
Analysis of non-identical and identical SNP alleles in haploid calls in a heatmap format amongst the genomes. Samples are listed according to the NJ dendrogram (shown on the left and top edges, along with population genomic data with k = 10). Heatmap scales are on the right side. Four genomes with a high proportion of missing data are also included, resulting in uniform lines on the heatmaps. **a** number of non-identical variants **b** number of identical variants. Note the high divergence of the B clade resulting in a higher number of non-identical SNPs compared to all other clades and the high uniformity in *A. orzyae* clades.

To further compare the various clades and the two newly-delineated mosaic groups, we also visualised non-identical and identical variants across all samples in a heatmap, following the order of genomes in the NJ dendrogram. As shown in Fig. 5, the clades and the subclades of the two biocontrol strains in Flavus B and Flavus A were resolved, based both on the very low amount of non-identical variants (Fig. 5a) and in the very high number of identical ones (Fig. 5b). Notably, the Flavus B clade’s numerous samples shared 1.5 to 1.7 million variants (see also Suppl. material 1) and the number of non-identical variants was characteristically low in this clade (mean values ~ 34,000) compared to the similarly large Flavus A clade. The identical variants in the latter amounted to approx. 1.4 to 1.6 million, but the non-identical variants had a mean number reaching ~ 140,000. In both cases, the biocontrol subclades of Afla-Guard and AF36 were highly uniform. Similarly, homogeneous groups of samples were observed in the Flavus A lineage, in Oryzae A-B and in Oryzae C-H.

The four isolates with a high proportion of unknown variants were retained in this study (Suppl. material 1) to ensure the most comprehensive overview on the two species. Interestingly, both the neotypes of *A. flavus* and *A. oryzae* were found to be closely related within the Oryzae C-H clade, with no signs of admixture. The reference strain for *A. flavus*, on the other hand, was nested within the Flavus A clade (= lineage IC) as observed in previous studies (Drott et al. 2020; Molo et al. 2022).

### ﻿Geographic distribution and niche specialization in the clades

**Figure 6.**
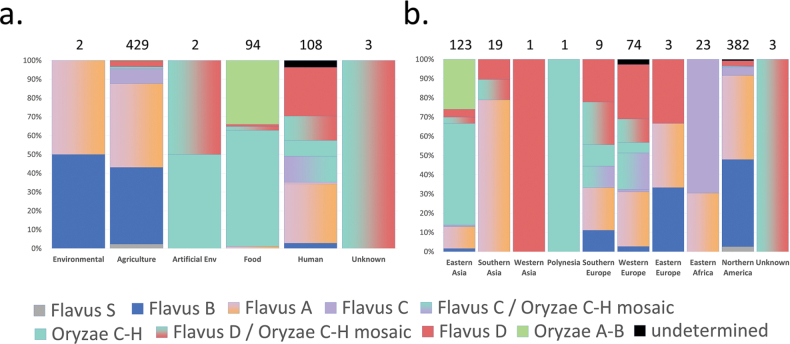
**a** Isolation sources of the 639 genomes and their placement into the various clades and mosaic groups **b** Geographic origin of the 639 genomes and their placement into the various clades and mosaic groups. Total number of genomes in each source type or geographic region is indicated above the stacked bar charts. Clades and mosaic groups are colour-coded. Note that the Human source contains 11 isogenic Flavus A genomes collected from a single patient (Suppl. material 2).

A total of 633 of the 639 genomes were assigned into clades or mosaic groups and further classified according to sampling source and geographic region. A significant proportion (429 genomes, 67.1%) originated from an agricultural setting and the Flavus A, B and C clades were dominant amongst them (Fig. 6a). Food and alcoholic beverage fermentations accounted for 94 samples, almost exclusively from the Oryzae A-B and Oryzae C-H clades. A total of 108 samples originated from human hosts, mainly from Flavus A, D and the two mosaic groups, while some genomes belonged to the Oryzae C-H clade. Other sample sources were natural environment (2 samples) and artificial substrates (2 samples).

Many isolates (382) originated in northern America. This region was dominated by Flavus A and Flavus B genomes, along with large number of isolates in Flavus C and D clades. Eastern Asian samples predominantly originated from food and beverage fermentations and belonged to the two Oryzae clades, as well as the Flavus A lineage. Western European samples predominantly represented the Flavus D and Flavus A clades, the two mosaic groups (mostly in the form of human samples), while a smaller number of Flavus B and Oryzae C-H samples were also found amongst them (in the latter case, these were human samples). Eastern Africa and southern Asia were represented by substantially smaller sample sizes and were dominated by Flavus C and Flavus A and by Flavus A samples, respectively.

## ﻿Discussion

In this study, we provide a phylogenomic and population genomic overview using all available short-read genome sequence data for *A. flavus* and *A. oryzae*. We applied an approach relying on reference-based variant-calling that enables comparing their genomes even in cases where high-quality assemblies are not available. The 639 genomes included in the current dataset significantly exceed the sample sizes used in previous genome-wide SNP phylogenies. For comparison, Drott et al. (2020) examined 94 genomes, Gangurde et al. (2024) included 346 and Hatmaker et al. (2025) examined 300. However, the current dataset was smaller than the 815 genomes analysed by Molo et al. (2022) using ddRADSeq, which samples only a fraction of the genome. That study was also focused on a more restricted geographic area.

Similar to Molo et al. (2022), our analyses showed that the *A. flavus* and *A. oryzae* reference genomes are interchangeable for population and phylogenomic studies using a reference-guided variant discovery pipeline. This fact has important implications in metagenomics as well. Most mycobiome studies perform read mapping against a collection of fungal reference genomes, often using combined reference datasets available from NCBI. This contains several *A. flavus* and *A. oryzae* references and, if no similarity-based clustering method is applied prior to read mapping (e.g. as applied in Yan et al. (2024)), reads from metagenomic libraries may map to both. This would indicate the simultaneous presence of both lineages even if only one is present. Similarly, several databases used for ITS metabarcoding were found to contain both *A. flavus* and *A. oryzae*. This may also result in unreliable mapping of amplicons and the simultaneous identification of both species. This may cause important uncertainties, especially in cases where the presence of both is not unexpected. For example, Peng et al. (2025) identified both species in some traditional solid-state fermentations, based on high-throughput amplicon sequencing.

Our comprehensive database on available sequenced strains and isolates of both species along with their collection metadata (Suppl. material 1) aimed to provide information for further studies, facilitating large-scale phylogenies and the correct placement of newly-sequenced genomes. However, apparent biases could be observed in the list of genomes, with samples from the USA over-represented in comparison with other geographic areas where *A. flavus* is a common agricultural problem. Another apparent bias in the current dataset was the presence of large clusters of the most used biocontrol agents, Afla-Guard and AF36. The presence of these highly similar genomes can bias population genomic algorithms, as shown here (Figs 1, 3). The African continent was clearly under-represented in the dataset and several described variants of the two species lack sequenced type or neotype strains. Samples from natural ecosystems were almost completely missing from the global dataset, with only two samples worldwide. Sampling efforts and sequencing novel isolates is paramount to understand the global diversity of these species. This was perhaps best illustrated by the fact that, in this study, while only three Central European samples were sequenced, they represented three distinct major clades: Flavus B, Flavus A and Flavus D. More sequences would certainly reveal more about the biogeography of the clades. It is especially important to note that the entire lineage II (that likely corresponds to the cluster named S_BG_, considered to be an interspecific mosaic) is missing from our present analyses as none of its members has been sequenced so far. There are also no available sequences for A. flavus var. parvisclerotigenus and, therefore, its placement and relatedness to *A. flavus* and *A. oryzae* remains unclear.

The clades delineated here based on NJ-based network, ML phylogeny and population genomic analysis corresponded to the previously described ones (in the case of *A. flavus*, originally referred to as populations) (Drott et al. 2020; Hatmaker et al. 2025) and the fact that *A. oryzae* was analysed together with the *A. flavus* genomes in a comprehensive phylogeny enabled an unprecedented overview of their clades’ relationships. Reconstruction of the genome’s evolutionary history recapitulated the existence of *A. flavus* evolutionary lineages 1A (= Flavus S), IB (= Flavus B) and IC (= Flavus A) and identified *A. flavus* populations that were intermixed with *A. oryzae* (Flavus C and D). This further supports the important role of genetic exchange and recombination in driving further clade diversification. For example, the two newly-delineated mosaic groups showed genomic ancestry intermediate between well-established clades of both species, such as Flavus D and Orzyzae C-H and Flavus C and Oryzae C-H. Ongoing genetic exchange and recombination may also confound phylogenetic inferences, which assume a strictly bifurcating evolutionary history. We showed that the mosaic genomes may be misplaced in Maximum Likelihood-based phylogenies (Fig. 4) and only the integration of network and population-genomic analyses can reliably reveal their admixed nature. Relatively high levels of introgression were also detected in the more well-defined clades, for example in Oryzae A-B and in the highly diverse Flavus A clade that was characterised by a highly heterogeneous within-clade structure (Figs 1–4). In the case of the latter, FastStructure identified two to three presumed populations with diverse forms of admixture amongst these, without clear subclades (apart from the biocontrol AF36 and its very closely-related isolates). Lineage IC (= Flavus A) appears to be the evolutionary cradle of *A. flavus*, harbouring more genetic and functional diversity (e.g. aflatoxin chemotypes) than any other species in section Flavi (Carbone et al. 2007; Moore et al. 2009). While introgression from Flavus B and Flavus D into Flavus A was evident (Figs 2, 3), Flavus B itself did not display such patterns. These patterns are reminiscent of the recent findings of asymmetric introgression into Flavus A observed in a substantial number of North American isolates (Molo et al. 2022) and of the low levels of admixture in Flavus B reported by Hatmaker et al. (Hatmaker et al. 2025). Furthermore, a recently published and subsequently withdrawn preprint described phylogenomic and population genomic analyses of more than 550 unpublished *A. flavus* genomes from China. These genomes remain inaccessible (Xie et al. 2023).

Our understanding of the genetic relatedness amongst agricultural, clinical and domesticated strains of *A. flavus* remains limited. The Flavus D clade, described recently as an *A. flavus* population of infectious human isolates, is related to Flavus C (Hatmaker et al. 2025) and was found to be much more closely related to *A. oryzae* clades than to Flavus C in our extended dataset that also included genomes of the koji fungi (Figs 1, 2). This raises the question of whether domestication and the colonisation or infection of the human host are related processes in section Flavi, similar to what has been observed in *Saccharomyces cerevisiae* baker’s yeasts. In this latter, widely used species, both non-admixed and highly admixed clades adapted to fermentation niches proved to be associated with isolates colonising and infecting humans, while wild clades in natural niches not (Peter et al. 2018; Loegler et al. 2025; Rácz et al. 2025). In the current *Aspergillus* dataset, human isolates represented a substantial proportion (~ 17%) of all genomes and these isolates predominantly grouped with the Flavus D and Oryzae C-H clades, along with the two mosaic groups related to these clades (and to Flavus C in the case of one mosaic group). The remaining human isolates fell almost exclusively within the Flavus A clade, including a clonal cluster of 11 samples from a single patient from the Netherlands (Buil et al. 2021). As expected, the Oryzae A-B and Oryzae C-H clades were highly enriched in isolates from solid state fermentations for traditional food products and alcoholic beverages and are considered domesticated (Watarai et al. 2019; Chacón-Vargas et al. 2021). Several hypotheses about the relationship between domestication and human infections may be proposed, but their evaluation will require further study. For example, domestication and frequent use could merely have promoted increased opportunities for human infections or, alternatively, domestication may have contributed pre-adaptive traits for the infectious groups of these fungi. This needs to be further elucidated by studies with broader sampling, thereby lowering the potential effect of sampling bias. Amongst the currently available clinical isolate genomes, Europe is vastly over-represented, while agricultural isolates are extremely low in numbers from the continent. This may obscure our understanding on the potential origin of clinical isolates.

It is also possible that a single earlier clade bifurcated into what are now predominantly clinical and food fermentation clades with contrasting evolutionary histories. Another important observation about the clinical isolates is that they are not only common in the Flavus D clade (Hatmaker et al. 2025), but also in the Flavus D/Oryzae C-H and the Flavus C/Oryzae C-H mosaic groups, where clinical samples are found almost exclusively, even in higher proportions than in Flavus D (Fig. 2). These findings indicate that genetic admixture may be a significant evolutionary force in the origin of the approximately 30 clinical genomes in these groups. Alternatively, the apparent lack of non-clinical strains in these mosaic groups may simply reflect sampling bias and increased sampling may later clarify whether admixture contributed to pathogenicity in humans.

The most striking observations made when comparing all available short-read sequenced genomes are related to the status of the koji fungus and its treatment in regard to the species delimitation of *A. flavus*. We found that the neotype strain of *A. flavus*, NRRL 1957, was placed in the Oryzae C-H clade closely related to the neotype strain of A. oryzae var. oryzae, namely RIB40. Similarly, Hatmaker et al. (2025) placed this genome in population D, which, in the present study, was divided into distinct clades and mosaic groups due to broader sampling and the inclusion of koji fungus genomes. This unexpected placement has major taxonomic implications, as it is impossible to split the members of the two *Aspergillus* species into any two monophyletic groups that: (1) would together encompass all samples and (2) contain only one of the neotypes in each. In other words, A. oryzae var. oryzae should not just be treated as a junior synonym of *A. flavus* because the koji fungus originated from within the diversity of *A. flavus*, but also because the very clade that contains its neotype already holds the neotype that represents *A. flavus* itself. A similar conclusion was reached in a preprint, based on phylogenomic analysis of 313 *A. flavus* and *A. oryzae* genomes during the review process of this current work (Drott et al. 2025). Furthermore, the koji fungus was split into two well-separated clades that did not form a single monophyletic group, suggesting at least two independent domestication events and very limited subsequent admixture from the Oryzae C-H to Oryzae A-B clades (Figs 2, 3). The Oryzae A-B consisted of two groups of apparently clonal genomes, both with very low genetic diversity (Figs 1, 5). The origin of this clade remains unclear as the number of variants it shared with other clades and mosaic groups (Fig. 5, Suppl. material 1) and its population genomic relationships (Fig. 3) placed it well-separated from others. Additionally, the larger clonal group, namely Oryzae A of Watarai et al. (2019) showed no sign of admixture. Importantly, based on two fully-assembled genomes and their structural differences, it has already been shown that there are differences in alpha-amylase gene duplications, in gene presence/absence and in high-impact mutations in biosynthetic genes in the two *A. oryzae* clades (Chacón-Vargas et al. 2021). These suggested independent domestication events.

Gell et al. (2020) constructed genetic maps, based on three *A. flavus* mapping populations to gain insights into the genomic organisation of *A. flavus* and how it differs from *A. oryzae* RIB40. They found an inverted reciprocal translocation between chromosomes 2 and 6 that was present in all six parental strains, which included strains from both lineages IB (= Flavus B) and lineage IC (= Flavus A), when these genomes were compared to *A. oryzae* RIB40. They hypothesised that the inverted reciprocal translocation event most likely occurred between chromosomes 2 and 6 in the RIB40 strain, potentially during the process of domestication. It is possible that domestication has led to additional translocation events, occurring independently and multiple times across different species, evolutionary lineages and populations; these are mostly undetectable using short-read sequencing and reference-guided variant discovery methods. Future work should include extensive *de novo* long-read sequencing and assembly of mosaic and non-mosaic genomes to determine whether structural variants are confounding evolutionary relationships within these groups.

These revised phylogenetic relationships have profound implications for several applied fields, including food fermentations and safety, animal nutrition and human clinical diagnostics, as well as metagenomics and metabarcoding. It also complicates efforts to align taxonomy with applied mycological concepts and practices in food technology and safety. A clearer, global overview of the genomic diversity and population structure of these fungi can help in supporting regulatory decisions and diagnostics, especially as agricultural *A. flavus* and *A. oryzae* strains are increasingly used as biocontrol agents against toxigenic strains (Lewis et al. 2019; Molo et al. 2019; Khan et al. 2021) and insect pests (You et al. 2024). Similarly, the koji fungus is widely sought after for at-home fermentations, gaining attention as a source for health-promoting foods and post-biotics (Choi et al. 2024; Seidler et al. 2024) and feedstuff supplements (Uwineza et al. 2024). These uses may facilitate the spread of several clades or mosaic groups into novel ecological niches and geographic regions.

## ﻿Conclusions

Here, for the first time, we analysed all available *A. flavus* and *A. oryzae* short-read genomes together. This joint analysis not only confirmed the origin of the domesticated form (*A. oryzae*) from *A. flavus*, but also revealed an unexpectedly complex population structure, with mostly well-separated clades interspersed with highly admixed mosaic groups. The distinction between these fungi as two separate species is complicated by the paraphyly of *A. flavus* when *A. oryzae* is excluded and by evidence of independent domestication events that gave rise to the koji fungus. Clarifying their taxonomic status will require thorough and, potentially, controversial decisions, but doing so would ultimately improve safety assessments in food production, provide information for biocontrol applications and increase the accuracy of automated metagenomic analyses. Our phylogenomic and population genomic results substantially altered the context in which the origin and diversity of *A. flavus* clinical isolates are understood, relative to recent work (Hatmaker et al. 2025). Human-infecting isolates are not merely enriched in a single distinct clade of *A. flavus* (Flavus D), but instead occur across a broader set of clades and mosaic groups that also include traditional food fermentation isolates of *A. oryzae*. Future studies should, therefore, consider domestication history when analysing genomic markers of pathogenicity.

As domestication and admixture can move accessory genes between lineages, the pangenome and a comprehensive analysis of structural variations in the genome is the logical next frontier. Although we did not analyse the pangenome of the 639 samples here, subsequent work should clarify which accessory genes in *A. flavus* mosaic groups may derive from *A. oryzae* and, thus, from domestication. These efforts could even adopt a super-pangenome framework, recently demonstrated in plant genomics where domesticated lineages and related wild species are subjected to an integrative comparative analysis (Khan et al. 2020). Such a framework will require expanded geographic sampling (notably, Africa), sequencing of previously described, but unsampled groups (e.g. lineage II) and inclusion of closely-related wild and domesticated species, such as *A. minisclerotigenes*, *A. parasiticus* and *A. sojae*. This has the potential to resolve the evolutionary origins of accessory genes important in virulence, secondary metabolite production or carbohydrate metabolism in domesticated and non-domesticated lineages and would clarify the roles of domestication and admixture played in shaping adaptation to specific niches, like the human host or food fermentation.

## ﻿Additional information

### Use of AI

No use of AI was reported.

### Adherence to national and international regulations

All the fungal strains used in this study have been legally obtained, respecting the Convention on Biological Diversity (Rio Convention).

### Author ORCIDs

Walter P. Pfliegler https://orcid.org/0000-0001-6723-4416

Bálint Németh https://orcid.org/0009-0004-4654-6073

Veronika Bodnár https://orcid.org/0009-0008-3241-8889

Tünde Pusztahelyi https://orcid.org/0000-0002-5495-6273

Ignazio Carbone https://orcid.org/0000-0003-2721-6656

István Pócsi https://orcid.org/0000-0003-2692-6453

### Data availability

The datasets generated during the current study are available in the FigShare repository (https://figshare.com/projects/The_clades_mosaic_groups_domestication_history_and_the_global_diversity_of_the_Aspergillus_flavus_oryzae_complex/260192) and in NCBI SRA (PRJNA1307175).

## Supplementary materials

Supplementary material 1 Additional figures on variant calling and analysis Data type docx https://binary.pensoft.net/file/1498701This dataset is made available under the Open Database License (http://opendatacommons.org/licenses/odbl/1.0/). The Open Database License (ODbL) is a license agreement intended to allow users to freely share, modify, and use this Dataset while maintaining this same freedom for others, provided that the original source and author(s) are credited.Walter P. Pfliegler, Bálint Németh, Veronika Bodnár, Tünde Pusztahelyi, Ignazio Carbone, István Pócsi

Supplementary material 2 List of strains and isolates with sequenced genomes Data type xlsx Explanation note All sequenced Aspergillus flavus and oryzae strains and isolates, and their metadata. https://binary.pensoft.net/file/1498702This dataset is made available under the Open Database License (http://opendatacommons.org/licenses/odbl/1.0/). The Open Database License (ODbL) is a license agreement intended to allow users to freely share, modify, and use this Dataset while maintaining this same freedom for others, provided that the original source and author(s) are credited.Walter P. Pfliegler, Bálint Németh, Veronika Bodnár, Tünde Pusztahelyi, Ignazio Carbone, István Pócsi
