# Supplementary material for: ﻿Global population genomics redefines domestication and clinical diversity in the Aspergillus flavus–oryzae complex
Source: IMA Fungus. 2025 Dec 23;16:e172343. doi: 10.3897/imafungus.16.172343 (PMC12780890; doi:10.3897/imafungus.16.172343)

**Supplementary File S1.** Additional figures related to variant calling.

Proportion of unknown variants and variant positions called as gaps in the cohort calling dataset in the form of box plots for each group. Mapping the *A. minisclerotigenes* and *A. parasiticus* reads to the NRRL 3357 reference resulted in a high proportion of callable sites despite their divergence (gap and ambiguity: 9.36% and 14.22% of total 3,469,698 called variant sites, respectively), these are not shown in the figure below. For the boxplots, linear quartile method was used, median is shown in the form of a darker line, the whiskers don't include the outliers (but show minimum and maximum values without outliers). Outliers appear as two types of points: outliers with >3×IQR (interquartile range) appear as a full red circle, suspected outliers with <1.5×IQR appear as an open circle. Outliers with more than 10% of unknown/gap variant sites are marked with their genome identifiers (four samples).


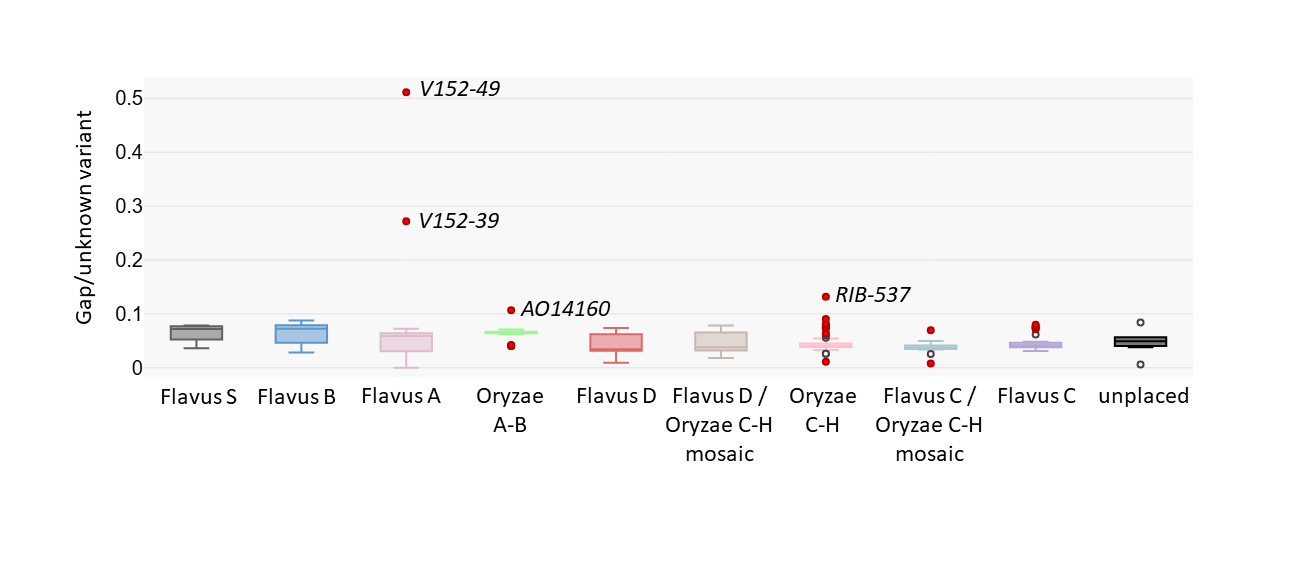


Below: Violin plot representations of the number of identical variants after comparing all possible pairs of genomes. The total counts of identical called variants in pairwise comparisons is shown for each possible group-level comparison, e.g. all Flavus S clade genomes were compared to each Flavus S clade genome, and to all Flavus B clade genomes, etc. These comparisons were made in the case of all nine clades and mosaic groups. Violin plots represent these comparisons, with box plots inside. Quartile method was linear, outliers are not included in boxplots (as described above). Note that in the Flavus A clade, samples VI152-49 and V-152-39 have a very high amount of unknown variants, resulting in low number of shared variants highly skewed violin plots. Each panel shows a single group of genomes (marked at the y axis) compared to all nine groups, y axis shows total number of shared variants. Where a clade or mosaic group is compared to itself, the plot is colored yellow.


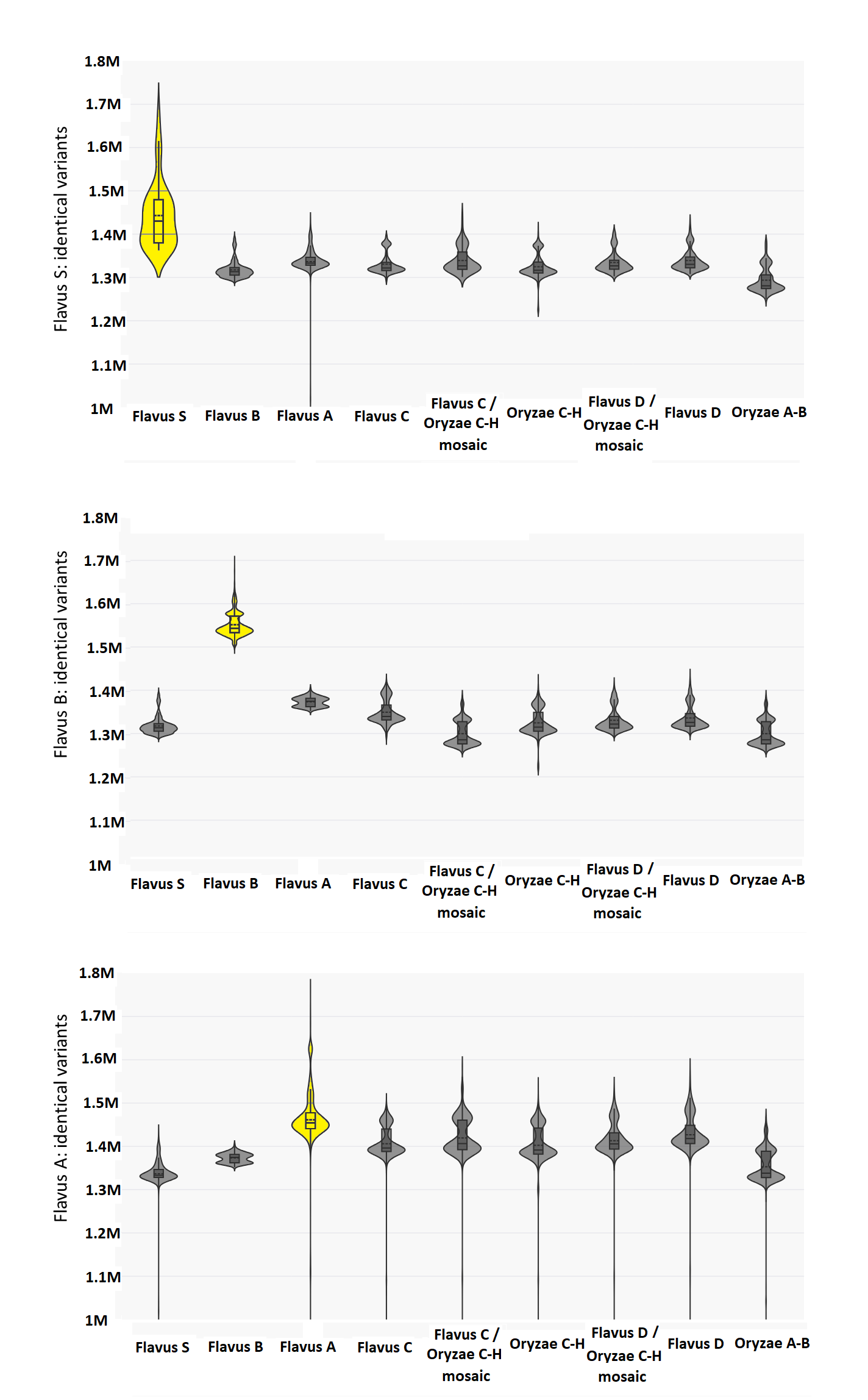


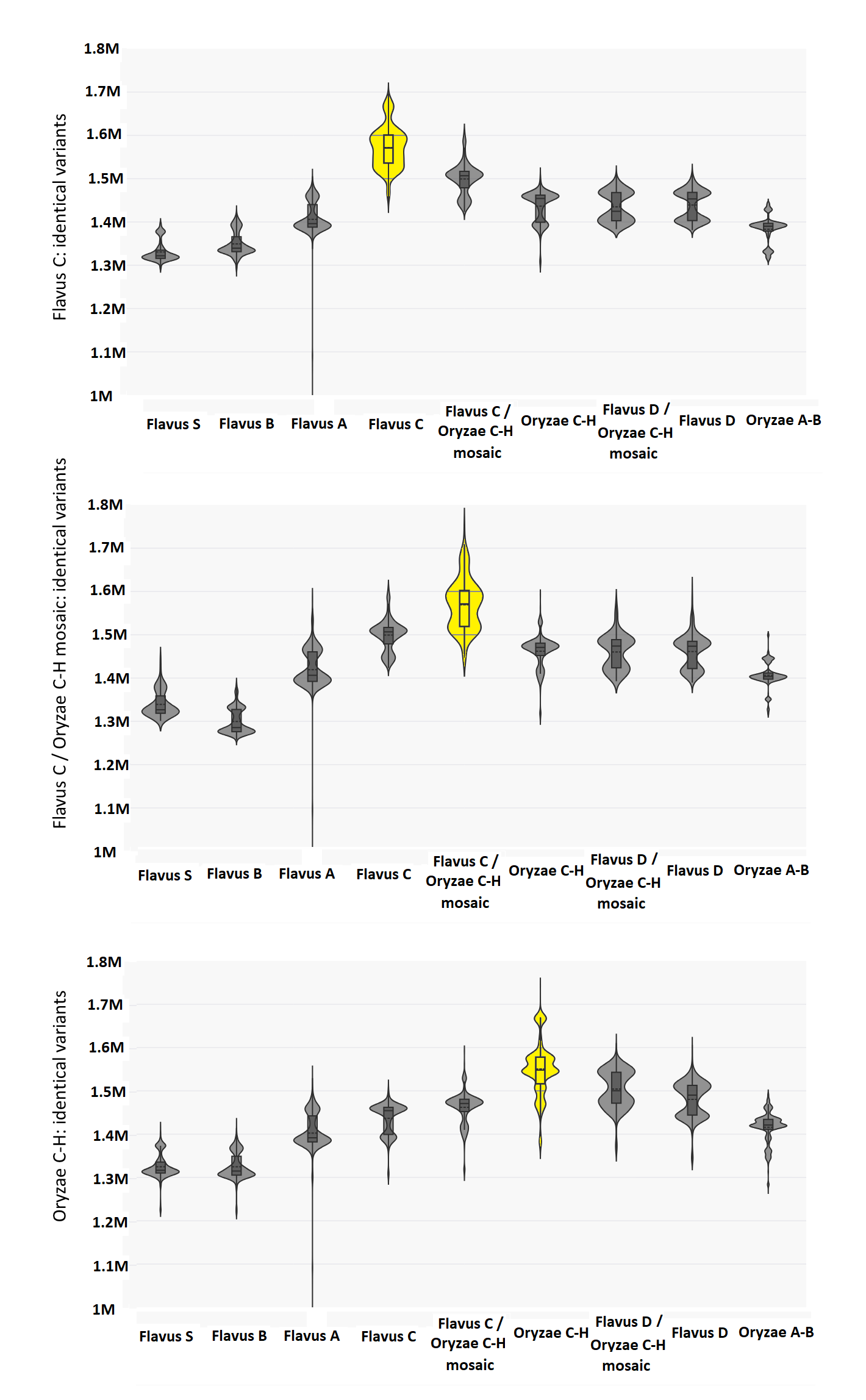

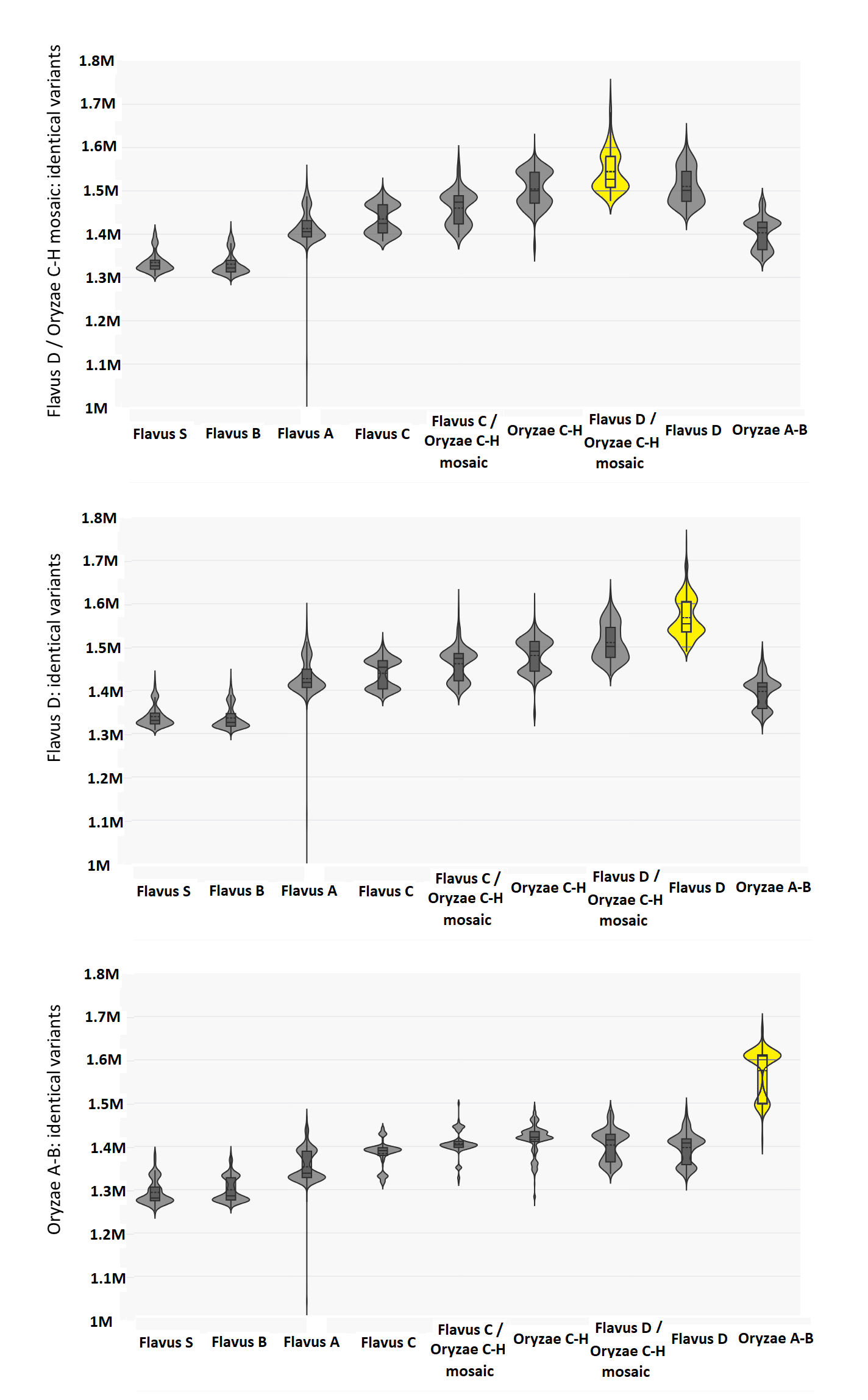

Supplement: Supplementary material 1 — Additional figures on variant calling and analysis [file imafungus-16-e172343-s001.docx]
